# Society Proceedings

**Published:** 1884-07

**Authors:** 


### SOCIETY PROCEEDINGS.

CHICAGO MEDICAL SOCIETY.

PUERPERAL FEVER—FRACTURES—THE GREATER TUBEROSITY OF THE HUMERUS, EXTENDING INTO THE BICIPITAL GROOVE.

A regular meeting of this Society was held on the evening of May 5th, 1884, when papers upon the above topics were read. The first, on "Puerperal Septicæmia and Prophylaxis of Puerperal Inflammations," was ably presented by Dr. G. F. Lydston, wherein he differed from a number of eminent writers on these subjects. The paper is some seventy odd pages in length, written on legal cap, and consumed the greater part of the evening's session. Below will be found a somewhat brief extract, for to dwell upon the merits of this lengthy article in detail would require more space than we can in this issue devote to it.

After reviewing with some vigor the discussion of "Puerperal Fever" that has appeared during the winter in an Eastern journal, and that of the New York County Medical Society, as well as the admirable essays upon the subject by Drs. Thomas and Garrigues, the writer asks, What is puerperal fever? To define this, he added, is a most difficult problem. Referring to the descriptions of Leishman, Hervieux and Lusk, how widely different are their definitions, described at intervals extending over a period of ten years. Barker states that puerperal fever is an entity. This definition, of course, is stripped of its superfluous and obscuring elements, all of which is denied by Lydston; and whether "septic" or "specific" puerperal infecion "be the assumed common cause in puerperal fever (*i. e.*, transposing his ideas), to assume that diseases which may occur from numerous and most diverse causes in the non-puerperal female should change and become suddenly specific" is illogical. To enumerate them all and their multiplicity would be an act of supererogation in this condensed report, nor shall we attempt doing so at this time.

The writer did not, however, wish to be understood as advancing any arguments in favor of ignoring the danger of septicæmia, but simply of offering a protest against what he was pleased to term the "septophobia" prevalent in some quarters. In other words, he defined his position as midway between the two extremes of practice. Through the medium of the placenta there is a constant interchange of nutritive and waste materials between the vascular systems of the mother and child. The former is far in excess of the latter, and is a physiological necessity. In this process of osmosis the fluids are chiefly towards the child. At birth, however, all this is changed, and the osmosis is in the direction towards the maternal circulation. The afferent current is checked, and the lymphatics and veins of the mother are more active than ever, as it is mainly through them that the retrograde metamorphosis of tissue occurs, which is the essence of physiological uterine involution; or, as is described later in the paper, the function of the lymphatics and veins of the puerperal woman is to remove those nutritive materials which by the removal of the fœtus have been rendered unnecessary. That a large amount of waste material is thus removed is evidenced by the peculiar character of the colostrum. This, rather than the patulous condition of the uterine sinuses, is the most important physiological circumstance favoring septic absorption, for with the products of retrograde metamorphosis of tissue we are likely to have absorbed the products of putrefactive changes. The so called "milk fever" may also be ascribed to this cause. It is not necessarily injurious, as experienced obstetricians have lately stated; and *Lusk* states that round micrococci are the necessary casual element of true septic fever. This, if it be true, agrees very well with the views just advanced. Lydston's belief is that milk fever is simply due to hyper-activity of the nutritive functions, resulting from the sudden introduction of an excessive amount of nutritive material into the circulation. It will be observed that the fever lasts only until the excess of material has had time for elimination, chiefly through the selective action of the mammary glands. Of course, it must be admitted that if products of putrefaction be also introduced, the rise of temperature and danger of eventuation in septicæmia will be proportionately increased. * * * Pale, weak, anæmic women are most liable to develop septicæmia, while more robust women if sick at all, are most likely to be attacked by some variety of local inflammation. Yet should the latter have the clinical characteristics of a typical case of the disease, the origo mali in the first case cannot be the same as that of the second, and the two forms will depend upon widely different causes, in spite of the theory of the common cause, "septic absorption." Hypothetical typical cases of "puerperal fever," "local inflammations," with moderate and quite intense fever, etc., were clearly illustrated and graphically described in a few succeeding pages of the paper; where one woman that has passed through labor is a victim to toxic infection, whilst the other, who is plethoric, is markedly predisposed to inflammation; the one, whose tissues do not want an abundance of nutritive pabulum; the other, whose tissues fairly crave supplies of new material, and who, practically speaking, is a huge sponge, ready for the absorption of any materials of an organic nature. These conditions of debility favor unhealthy and putrescible secretions.

The writer was inclined to believe that septicæmia may occur from the absorption of fluid from which bacteria are absent; sepsin in its results differs not at all from snake virus, and its destructiveness depends in a great measure upon local and constitutional conditions in the affected individual. In "pyemia," however, which is but one grade of "septicæmia," bacteria must necessarily be present. In certain cases of mild febrile attacks following child-birth, it may be simply a traumatic fever. This, while often due to septic absorption, may also be due to nervous influences, or traumatic fever resulting from nutritive disturbances (as shock), which is due to excessive reaction following nervous depression. Shock also may be due to the use of instruments. Malarial fever may also come on in the puerperal state. Certainly, then, septic infection may not be present in any of these instances, and yet the case be diagnosed as septicæmia. In certain cases of septic infection following labor, we have diffuse suppurating abscesses, differing little, if any, from "pyemia" that may follow surgical operations; but the characteristic temperature curve is exceptional in the former, which is present in "pyæmic" fever. This form of puerperal septicæmia is sometimes very chronic, resulting in gluteal abscesses; and even secondary suppurative processes have been found (by himself) at the autopsy in the liver and kidneys. The causal element of the phases of the diseases usually termed respectively pyæmia and septicæmia, is the same, but the former is a misnomer. In one we have the formation of thrombi, which contain micrococci, and which, becoming detached, enter the circulation until they lodge and form new foci for suppuration, etc., while in the other no thrombi are formed, or if formed become so rapidly and thoroughly disintegrated that secondary suppurations do not result. Comparative illustrations were then given why metastatic abscesses cannot result from dissecting a wound which is septicæmic. In a general way, the formation or non-formation of thrombi depends upon the intensity of the infection and the local and constitutional conditions present. Metastatic abscesses are quite exceptionally seen in the puerperal state. This may be due, in part at least, to the fact that the patients are so speedily destroyed by the intense infection characteristic of puerperal septicæmia. Thomas goes to the extreme of attributing all puerperal disorders to the absorption of septic poison; and, in further quoting that author, according to the writer, it matters not whether the disease be a phlebitis, cellulitis, lymphangitis, or peritonitis, the essence of the disorder is the absorp- of poison into the blood of the puerperal woman through some solution of continuity in the genital tract. * * * How about exposure as a cause? Phlebitis following labor may give rise to the disease known as phlegmasia dolens, and may result from trauma, especially if exposure be superadded; or it may arise from simple thrombosis, occcuring independently of bacteria or septic processes, in the same manner as cellulitis may arise. * * * A "locus minoris resistentiæ" may be afforded; and the woman being especially susceptible to cold and depressing influences of all kinds, it remains but for an exposure to a draught of air to light up an inflammation of the uterus, constituting a metritis, or a pelvi-peritonitis, or cellulitis. The simple passage of a sound into the non-pregnant uterus has been known to produce pelvic inflammation, severe cellulitis, etc. A rapidly fatal general peritonitis has also been caused in the puerperal woman by simple extension from the pelvic peritoneum. In these cases, if septicæmia occur at all, it is secondary to or a complication of the inflammatory affection. Considerable space was allotted in the paper to illustrate the probably fallacious reasoning of those who affirm that septicæmia is the term including the "many synonyms" of disease to which the puerperal woman is liable, when Parry's classification of these disorders was given, the principal feature of which consists in fever, with its various concomitants. Now, as this is also somewhat dogmatic and arbitrary in its division, yet it is often sufficiently practicable to enable us to recognize the existence in different cases of the affections named. Lydston, however, suggested a modification of Parry's classification which he thinks will enhance its accuracy. Regarding his suggestions, we will mention that only two should be classed "specific," and pyemia should be omitted altogether.

Before closing the review of this portion of the article, we wish to state again that Dr. Lydston does not deny that puerperal peritonitis is undoubtedly often septic in origin; but he emphatically insists that it is not always so, and that there is not a characteristic "septic peritonitis." * * * Regarding the conversion of the poison of the exanthemata into a "specific puerperal poison," the writer states it is absolutely impossible, for the reasons explained in the paper. The "unity" of the "materies morbi," the apparent interdependence of the poisons of different diseases, were to some extent forcibly impressed by the writer's citation of numerous cases and the variations dependent upon sanitary surroundings, etc. Considerable space was devoted to the diphtheria of puerperal wounds and puerperal erysipelas. The former, happily, is not of very great frequency, and it has the possibility, or a strong probability, of parasitic origin along the genital tract. The latter disease, however, may be located remote from the genitals, the same as in non-parturient women, a number of instances of which were given. Some cases of erysipelas were also given which could be traced to no other cause than cold and exposure to dampness, apparently independent of possible contagion.

Regarding prophylaxis of the puerperal diseases, added the writer, bi-chloride of mercury, antiseptic pads, and injections, with bare walls, floors, and other surroundings needed in small pox and typhus fever, are not a necessity in the lying-in chamber. In private practice, almost non-interference can be carried out, but under the present system of hospital construction this plan can rarely be so absolute.

Several pages were devoted to giving his experience while resident surgeon at Charity Hospital, New York, at Maternity Hospital, etc., and statistics which, compared with those given by Dr. Garrigues, do not well correspond with the latter.

From August, 1880, to December, 1880, there were confined in Maternity proper 50 selected cases; of these, five, or 10%, died of puerperal metritis, septicæmia, or peritonitis. In the waiting wards during the same months, there were confined 72 cases, also selected; of these, six died, only three of which, or about 4%, died of septic infection. During October and November, the writer delivered in the waiting wards alone 25 women, of whom one died, and she of extensive heart lesions and pulmonary œdema. During these same months, 30 out of the 50 cases cited from the Maternity records, five fatal cases occurred, making a mortality for the two months of 16⅔% from septicæmia at the Maternity proper.

In 1875, the first year of the occupancy of the Maternity Hospital, there were 570 deliveries, with a mortality of fifteen, or 2.67%. In 1881, the mortality fell to 2.36%; in 1877, it rose to 6.67%, the reasons of which were elaborately dwelt upon.

During the service of Dr. B. Wood at the Maternity, of 68

cases there were three deaths from all causes. In the next two months, of 62 cases three deaths occurred, one of which was due to septicæmia. The grand total for the waiting wards and pavilions from August, 1880, to April, 1881, was 202 cases, with a mortality of twelve, only five of which, or a little more than 2½% were due to septicæmia, or puerperal fever. Of 130 cases, the writer stated, the let-alone plan of treatment was more rigid than his own, vaginal injections not being allowed. From April, 1881, to April, 1882, 423 cases were delivered, with but two deaths from puerperal fever. The treatment of this series was also non-interference. On the other hand, the writer stated, he has seen the temperature rise and pelvic inflammations follow the use of frequent vaginal injections; and cases which may eventuate in puerperal septicæmia and lead to a fatal issue would often do well and make a perfect recovery if left alone and kept quiet and free from curious visitors. As for prophylactic intra-uterine injections, the man who uses them will sooner or later come to grief in making too general use of this procedure.

"Placental compression," or Crede's method, of "pressing it out," is almost invariably absurd and pernicious. No more harm or serious result is likely to arise at the present time from a few minutes retention of the placenta than there was centuries before Crede was ever heard of. Without wishing to underrate the importance of freeing the womb from putrescible substances, the writer deplored the practice of inserting one's hand into the uterine cavity, seeking for an imaginary piece of placenta or for portions of secundines of microscopic size.

Another, yet more clear, illustration of the advantages of the non-interference plan of treatment in obstetrical practice was cited while the writer acted in the capacity of Resident

Surgeon of the New York State Hospital for Emigrants, where the deaths occurring from septicæmia were at the minimum. A year since a so-called "reform" was instituted there in the emigration service; the prophylactic injections and complicated manipulations were introduced, and with the direct and immediate effect of increasing the mortality rate, which became alarmingly high. Simultaneously, upon Ward's Island, there were present a large number of Russian Jewish refugees, who were filthy and mentally despondent. Upwards of ninety of these women were delivered without a death. There were numerous forceps deliveries, but there was absolute non-interference in the after-treatment. Here was a good opportunity to compare fancy midwifery results with the let-alone method.

*Primary Operation for Perineal Lacerations.*—In severe lacerations the primary operation is very essential, and often productive of the best results; but the tendency is towards too much routine practice in this respect, especially in hospitals. Those of less degree will heal of themselves. Chromated catgut is probably the best material for sutures to be used in deep and extensive lacerations which it is desirable to repair at once, and thus diminish the surface for septic absorption; but if a laceration extend completely into the rectum the tendency is to the secondary in preference to the primary operation. Meanwhile, cleanliness and iodoform are the most important elements of treatment. In some cases the slight fever resulting from the sutures is likely to pervert the secretions and convert them into a favorable nidus for the development of bacteria. In general, the operation is very successful if properly performed. Instrumentation or passage of a catheter at frequent intervals is unnecessary. A bed-pan should be used, and a stream of iodized water from a fountain douche allowed to flow over the vulva during urination. Iodine is a more efficient deodorizer than carbolic acid, being destructive to bacteria in a strength of 1 to 5000. Bichloride of mercury, if used, should be in very dilute solutions, and in view of its chemical action in forming albuminates its superior usefulness may be questioned. A few other general suggestions for this accident closed the succeeding few pages upon the primary operation.

*Injections During Labor.*—This is recommended by some of our antiseptic extremists.

Lydston's remarks condensed were—they are not only useless, but injurious, and all unnecessary manipulations, as introduction of the hands frequently within the rima vulvæ is prejudicial to the safety of the patient; and only that which is consistent with careful watching of the case should be attempted.

*Tardy Delivery.*—The use of the forceps at the second stage, and care in changing of linen of the patient, first by warming it, also the bed-pan before placing it under a patient, received full attention by the writer's pen.

*Administration of Ergot.*—This drug is undoubtedly prophylactic in puerperal diseases, for it produces uterine contraction and hastens involution. In fuller subjects and after severe or instrumental deliveries, give small doses of the drug with quinine for a week or ten days. Hour-glass contraction should be carefully guarded against before the placenta has been expelled, although this condition is rare. After labor, a pad of oakum placed over the vulva is much neater than a napkin. The potency of germs and the value of antiseptics are well appreciated, but the woman should now be interfered with as little as possible. We should regard labor as a physiological process rather than as pathological. Women are to-day entitled to the same natural advantages that they were long before syringes, antiseptics, and microscopes were ever dreamed of, although when fetor or suppression of the lochia indicates danger, intra-utrine injections and other hygienic measures are demanded; and in these severe cases a glass tube after being introduced may be left in situ until danger is passed. Even constant irrigation may be advisable. Jones, of St. Paul, has recently reported several interesting cases in which they were useful. Sloan recommends suppositories of iodoform and eucalyptus. Curetting would not be proper except where portions of secundines were known to be left. The success, also, of the iodine treatment in typhoid fever ought to be a good basis for its use in septicæmia. Also antipyretics—as the salicylate of iron is almost specific in its action, this will often rapidly lower the temperature where quinine has failed. Then a judicious use of morphine and stimulants, with good nourishment, constitute (generally speaking) the proper management of puerperal septicæmia. The hot vaginal injections to be effectual should be given through a cylindrical speculum. The faradic current was spoken of as one of the best remedies to induce resolution of pelvic exudation. * * * General peritonitis complicated by septicæmia is the most fatal disease to which the puerperal woman is liable, the vital powers being overwhelmed by a most powerful poison. The treatment suggested for septicæmia comprises the measures already stated, in combination with very free use of opium or morphine or stimulants. Hot applications over the abdomen, or perhaps cold, may be a useful measure in this fatal malady and the support of the system in its efforts to rid itself of the poison already absorbed. About six pages of goodly advice to our esteemed bacteriophobic friends in the East, and to those having direct charge of Maternity Hospital, eloquently closed what probably is one of the most comprehensive papers upon a single topic ever presented to this Society. Many valuable formulæ and interesting points in this review have been unavoidably omitted, although their merits may receive further attention at some future time.

Fracture of the greater tuberosity of the humerus, extending into the bicipital groove, was the subject of a paper read by Dr. F. C. Schafer, who simultaneously presented the case of a man 55 years of age suffering from this rare form of fracture. As the paper is not at our command, the following abstract is made from memory. Two years ago, the patient received an injury to his shoulder by falling forward from a height of only about three feet, resulting in fracture of this uncommon variety. The various fractures and dislocations of the joint were enumerated, and the characteristics of this lesion were given as follows: A flattening of the shoulder and an increase in its antero-posterior diameter, will at first suggest that there is a dislocation to deal with. This flattening is caused by the drawing backward of the fragment of bone by the attached muscles and consequent crowding forward of the head of the humerus. The displacement leaves a deep sulcus in place of the bicipital groove. However, crepitation confirms the diagnosis. After the subsidence of the inflammation consequent to the injury, there was partial fibrinous anchylosis of the joint, which was with great difficulty broken up. At the present time, upward and backward extension of the arm is but slightly limited, as was demonstrated by the mobility of the limb. The alteration in shape of the upper part of the bone is evident on palpation only, and is barely perceptible to the eye, even when we attempt to manipulate the joint. The patient being somewhat fleshy, precludes any exterior signs of deformity. There has been almost perfect recovery resulting from the form of treatment devised.

In conclusion, the speaker cited several cases of this variety of fracture, in which the diagnosis had been confirmed by autopsy. Perhaps not more than ten cases of this fracture are on record. The patient was then examined by the members present, and its peculiarities informally discussed.

Owing to the late hour, and the fact that a number of members experienced surgeons and obstetricians had gone to Washington to attend the American Medical Association, the discussion of the papers was postponed until a future meeting.

A motion prevailed that the Society do adjourn.

